# Interpretation of the absorbed constituents and pharmacological effect of Spica Schizonepetae extract on non-small cell lung cancer

**DOI:** 10.1371/journal.pone.0248700

**Published:** 2021-03-17

**Authors:** Shuai Wang, Xinxin Yang, Wei Wang, Yunkun Zhang, Tianjiao Li, Lin Zhao, Yongrui Bao, Xiansheng Meng

**Affiliations:** 1 College of Pharmacy, Liaoning University of Traditional Chinese Medicine, Dalian, China; 2 Liaoning Multi-dimensional Analysis of Traditional Chinese Medicine Technical Innovation Center, Dalian, China; 3 The Second Affiliated Hospital of Dalian Medical University, Dalian, China; Duke University School of Medicine, UNITED STATES

## Abstract

As a traditional Chinese medicine (TCM) with a usage history of over 2,000 years in China, Spica Schizonepetae possesses definite clinical activity in the treatment of non-small cell lung cancer (NSCLC). However, its active ingredients and mechanism of action remain unclear at present. The further exploration of its active components and underlying mechanism will provide a basis for the development of candidate anti-tumor drugs. Our previous study explored the chemical constituents of Spica Schizonepetae extract (SSE). On this basis, molecular networking technology was applied in analyzing the QTOF-MS/MS data of rat plasma after intragastric administration of SSE using the GNPS database platform. A total of 26 components were found, including 9 proterotype components and 17 metabolites, which revealed the potential active ingredients of SSE. Later, the Lewis lung cancer mouse model was established, and the inhibition rate and histopathological sections were used as the indicators to investigate the anti-tumor effect of SSE, whereas the body weight, survival rate, thymus index and spleen index served as the indicators to explore the pharmacological effects of SSE on improving mouse immunity. The results showed that SSE had comparable anti-tumor efficacy to cisplatin, which enhanced the immunity, improved the quality of life, and extended the survival time of lung cancer mice. Furthermore, human A549 lung tumor cells were selected to explore the mechanism of SSE in treating NSCLC based on cell metabonomics. After data mining by the MPP software, 23 differential endogenous metabolites were identified between SSE and tumor groups. Moreover, results of pathway enrichment analysis using the MetaboAnalyst 4.0 software indicated that these metabolites were mainly enriched in four metabolic pathways (p < 0.1). By adopting the network pharmacology method, the metabolic pathways discovered by cell metabolomics were verified against the ChEMBL, STITCH, UniProt and TCGA databases, and differences in the underlying mechanism between cells and humans were found. It was proved that SSE affected the metabolism of purine, arachidonic acid and histidine to exert the anti-tumor efficacy. Furthermore, the multi-target, multi-pathway, and immunoenhancement mechanism of SSE in anti-tumor treatment was revealed, which provided a scientific basis for new drug development and the rational application of Spica Schizonepetae in clinic.

## 1. Introduction

Lung cancer is the malignant tumor with the highest morbidity and mortality rates in the world [1], among which, non-small cell lung cancer (NSCLC) accounts for 80%-85% of all lung cancer cases. Most patients are diagnosed at the advanced stage and are linked with dismal prognosis, with the 5-year survival rate of less than 15%. Spica Schizonepetae is first recorded in the Shennong’s Classic of Materia Medica, an ancient classic book in the traditional Chinese medicine (TCM). It possesses various pharmacologica activities, such as anti-tumor, anti-inflammation, analgesic, anti-virus, and bacteriostasis [2–4]. In clinical research, Spica Schizonepetae or the ancient Chinese medicine formulas with Spica Schizonepetae as the main medicine (like Zhisou powder, Yinqiao powder and some empirical formulas), have also been used to treat lung cancer and the related diseases [5].

In the preliminary experimental study, the UPLC-QTOP-MS technology is employed to comprehensively analyze the chemical components of Spica Schizonepetae extract (SSE), and altogether 31 chemical components are identified, including luteolin, hesperidin and other flavonoids, caffeic acid, protocatechuic aldehyde and other organic acids, as well as ursolic acid [6]. These compounds have been reported to exhibit definite anti-tumor activity [7–9]. Spica Schizonepetae is a clinically used anti-tumor TCM herb, but its active ingredients, pharmacological effects and mechanism of action remains unclear so far. Therefore, clarifying its active components, efficacy and underlying mechanism will provide a basis for its new drug development and rational clinical application.

In line with the theory of serum pharmacochemistry, only compounds in the bloodstream may probably become the effective constituents [10]. In this regard, it is a good way to explore the effective components of drugs from the perspective of compound absorption into the blood, especially for TCM with complex chemical components. Most compounds in the body will develop further reactions, such as oxidation reaction, reduction reaction, glucuronidation, sulfation and methylation to produce corresponding metabolites, thus exerting a therapeutic effect or being excreted from the body [11,12]. As a result, the analysis of blood components, especially the accurate analysis on related metabolites, has become the key to explore the effective components.

Molecular networking is a kind of tandem mass spectrometry (MS/MS) data organizational approach recently introduced in the fields of drug discovery and medicine [13]. In particular, the open access of the Global Natural Products Social Molecular Networking (GNPS) database since 2014 has brought great convenience for correlation processing and the identification of related compounds. According to the secondary fragments formed after entering the high resolution mass spectrometry LC-MS/MS, compounds with similar structure will produce similar MS/MS fragment ions under the same conditions [14]. Typically, the GNPS database automatically associates these similar compounds through the computer algorithm [15], and realizes the association, identification and visualization of blood components and their related metabolites under the assistance of software (such as Cytoscape).

In this study, the molecular networking technology based on LC-MS/MS was utilized to analyze the components of SSE absorbed into the blood of rats, so as to identify the active components with potential therapeutic effects. In addition, the Lewis lung cancer mouse model was established to evaluate the efficacy of SSE in the treatment of NSCLC, from the physiological and pathological perspectives, including tumor inhibition rate, survival time, body weight (BW), thymus index, spleen index and histopathological section. On this basis, cell metabolomics was adopted in combination with network pharmacology to reveal the mechanism of its efficacy, which laid an experimental basis for the new drug development and rational clinical application of Spica Schizonepetae in the treatment of lung cancer.

## 2. Materials and methods

### 2.1. Materials and reagents

Spica Schizonepetae was purchased from Hebei Anguo Pharmaceutical Wholesale Market and authenticated by Professor Xu Liang (Liaoning University of Traditional Chinese Medicine, Liaoning, China). The SSE was produced at our laboratory by 75% ethanol reflux extraction and purified using the HPD400 macroporous resin according to our previous research process [6], with the average extraction ratio of 3.72% and the relative standard deviation (RSD) of 1.95% (*n* = 3). Methanol and formic acid (MS grade) were acquired from Merck (Darmstadt, Germany). Purified water was provided by Hangzhou Wahaha Group Co., Ltd.

### 2.2. Cell lines

The murine LLC cell line was purchased from Wuhan Boster Biological Technology., LTD. The A549 human NSCLC line was obtained from Saibaikang Shanghai Biotechnology Co., Ltd. These two cell lines were both cultured in the Roswell Park Memorial Institute medium (RPMI-1640) supplemented with 10% fetal bovine serum (FBS) and 1% penicillin streptomycin antibiotic mixture, and incubated in a humidified incubator under 5% CO_**2**_ and 37°C conditions.

### 2.3. Animals

A total of thirty-six male SPF Sprague-Dawley (SD) rats (weighing 180–220 g) and forty-eight male C57BL/6 mice (weighing 18–22 g) were acquired from the Liaoning Changsheng Biotechnology Co. Ltd (Liaoning, China; License Key: SCXK (Liao) 2015–0001). The experimental protocol was approved by the Animal Ethical and Welfare Committee of Liaoning University of Traditional Chinese Medicine. All experiments were conducted in strict accordance with the Guide for the Care and Use of Laboratory Animals of Liaoning University of Traditional Chinese Medicine (131/2010). All animals were housed in the animal room (25 ± 2°C, 60 ± 5% relative humidity) with a 12 h 12 h dark/light cycle.

### 2.4. Chemical components absorbed into rat plasma

#### 2.4.1. Collection and preparation of plasma samples

After acclimatization for 7 days, the SD rats were randomly divided into control group, drug group 1 and drug group 2, with 12 mice in each group. Rats in drug treatment groups were orally administrated with SSE solution (1.692 g/kg/d crude drug/BW/day) twice a day for seven consecutive days, while those in control group were given an equivalent amount of purified water. Plasma samples from drug treatment groups were collected after the last administration for 1 and 2 h, respectively, and isolated by centrifugation at 3500 rpm for 10 min at 4°C. Afterwards, an aliquot of 200 μL plasma sample was added into a clean centrifuge tube, and then 1000 μL methanol: acetonitrile solvent (1:1, V/V, 4°C) was added. Later, the mixture was subjected to vortexing for 2 min, ultrasonic extraction for 1 min, refrigeration at -20°C for 10 min, and then centrifugation at 12,000 g for 15 min at 4°C to precipitate protein. Then, 1100 μL supernatant was collected and transferred into a centrifuge tube for vacuum drying. The residues were reconstituted in 50 μL methanol: acetonitrile solvent (1:1, V/V), followed by vortexing for 2 min, ultrasonic extraction for 1 min, and centrifugation at 12,000 g for 10 min at 4°C. Afterwards, the supernatant was collected and transferred into an autosampler vial for following UPLC-QTOF/MS analysis.

#### 2.4.2. UPLC-QTOF-MS analysis

Plasma samples were analyzed on an Agilent-1290 UPLC system coupled with an Agilent-6550 QTOF mass spectrometer. An Agilent poroshell 120 column (2.7 μm, 100 mm × 4.6 mm, Agilent Technologies, Inc., USA) was utilized for chromatographic separation, with the column temperature being maintained at 30°C, the flow rate of 0.8 mL/min, and the injection volume of 3 μL. In further mass spectrometry in the positive ion mode, the mobile phase was constituted by 0.1% formic acid water (A) and acetonitrile-methanol (95:5, V/V) (B), and the gradient elution process was as follows: 0~5 min, 5→20% B; and 5~60 min, 20→100% B. In the negative ion mode, the mobile phase consisted of water (A) and acetonitrile-methanol (95:5, V/V) (B), and the gradient elution program was shown below: 0~17 min, 5%→40% B; 17~22 min, 40%→65% B; 22~45 min, 65%→95% B; and 45~50 min, 95%→100% B.

Both the positive and negative ion modes were used for mass detection. The source parameters were set as follows: Capillary voltage (Vcap), 4000 V; drying gas temperature, 250°C; drying gas flow rate, 13 L/min; nebulizer pressure, 45 psig; fragmentor voltage, 125 V; sheath gas temp, 350°C; sheath gas flow, 11 L/min; and acquisition rate, 1.5 Spectra/s. The MS data were collected in the full scan mode from m/z 100–1000, and the MS/MS analysis was acquired in the auto MS/MS mode with 40 eV.

#### 2.4.3. Data processing

First of all, the collected AUTO MS/MS data were imported into the ProteoWizard software separately, then the.d type files were converted into the.mzXML files and uploaded using the Filezilla software online to Global Natural Products Social Molecular Networking (GNPS) (**http://gnps.ucsd.edu**), a molecular networking and data-sharing web-based platform. Afterwards, the MASSBANK and MASSBANKEU databases were selected. The following parameters were set: precursor ion mass tolerance, 0.02 Da; fragment ion mass tolerance, 0.02 Da; minimum matched fragment ions, 2; min pairs cos, 0.7; and the data were analyzed. Afterwards, the GraphML data in each group were downloaded for Cytoscape analysis. The Difference Merge function of Cytoscape 3.7.2 software was employed to identify the differential compounds between control plasma and the administrated plasma. In combination with the 31 chemical components in SSE identified in our previously published paper [6] after excluding other interfering components, the protoplasmic components and their related metabolites were identified, and later a molecular network was established.

### 2.5. Anti-lung cancer activity of SSE in C57BL/6 mice

#### 2.5.1. Lewis lung cancer model and treatment

The LLC cells at logarithmic phase were harvested, suspended (1×10^**7**^ cells/mL) in 0.2 mL phosphate-buffered saline (PBS), and carefully implanted intradermally into the right axila of C57BL/6 mice, respectively. At one day after LLC inoculation, the mice were randomized as control group, model group, drug group (0.144 g/kg crude drug/BW) and cisplatin group (4.55 mg/kg), respectively, with 12 mice in each group. Mice in dose groups were given oral administration with corresponding drugs at a dose of 0.2 mL/20 g BW daily for 14 days, whereas those in control and model groups were given oral administration with 0.2 mL of 0.9% NaCl on the same schedule. The BW and survival of mice were recorded weekly. Typically, the survival rate was calculated according to the following formula: survival rate = number of survival mice/original number of mice × 100%. On the 15th day, the mice were sacrificed, then the tumor, spleen and thymus were immediately removed, and their weights were measured on the balance. The tumor inhibition rate was calculated as follows: inhibition rate = [1 − (tumor weight in each drug treatment group/average tumor weight of model group)] × 100%. The spleen and thymus indices were evaluated by the formula below: spleen or thymus weight (g)/body weight (g) × 100%.

#### 2.5.2. Histological examination

All the tumor tissues dissected from mice were weighed, fixed with 10% neutral phosphate-buffered formalin for 4 h, and processed for paraffin embedding in accordance with the standard histological procedures. Thereafter, the embedded tissue was serially sliced into the 5-μm sections, and then paraffin-embedded sections were deparaffinized in xylene, rehydrated with gradient ethanol, and stained with hematoxylin and eosin (H&E).

### 2.6. Mechanism of action based on cell metabolomics

#### 2.6.1. A549 cell viability assay

100 μL A549 cells (1×10^**5**^ cells/mL) at logarithmic phase were inoculated into each well of the 96-well plates. At 12 h later, the culture solution was discarded. Then, 150 μL medium supplemented with SSE at the concentrations of 11.0, 13.0, 15.0, 17.0, 19.0, 21.0 and 23.0 μg/mL was added into each well of the plate (*n* = 5) to culture for another 24 h. Afterwards, 100 μL culture medium supplemented with 10% CCT-8 was added in dark into each well. At 2 h later, the absorbance value (OD) was measured at 450 nm. The survival rate and IC_**50**_ value were also calculated.

#### 2.6.2. Cell sample treatment

A549 cells at logarithmic phase were inoculated into the two groups of culture bottles (25 cm^**2**^ on average), which were divided into a tumor group and a dose group, with 8 bottles in each group. When the cell density reached 10^**7**^ cells/mL, the dose group was cultured with the culture solution containing 45 μg of 17.0 μg/mL SSE per bottle, whereas the tumor group was given the same amount of culture solution without drug. After 24 h, the culture solution was discarded, and 1 mL of the 80% methanol (-20°C) was added into each bottle. The adherent A549 cells were quickly scraped off with a cell scraper. Later, the cell suspension was collected and quenched at -80°C for 30 min. At a 20% power of the ultrasonic crusher, the cell suspension was subjected to 5 min of ultrasonic crushing in the ice bath. Subsequently, 200 μL suspension was collected from each bottle and treated with the protein removal column. After elution with the pre-cooled methanol at a 3-fold volume, the column liquid and eluent were collected and mixed for subsequent analysis.

#### 2.6.3. UPLC-QTOF-MS analysis

Cell samples were analyzed on an Agilent-1290 UPLC system coupled with an Agilent-6550 QTOF mass spectrometry. Additionally, an Agilent Eclipse Plus C_**18**_ column (1.8 μm, 50 mm×3.0 mm, Agilent Technologies, Inc., USA) was used for chromatographic separation. The mobile phase consisted of ultrapure water containing 0.1% formic acid (A) and methanol (B), and the flow rate was set at 0.4 mL/min. The gradient conditions of the mobile phase were as follows: 0~5 min, B 5%→60%; 5~10 min, B 60%→100%; 10~15 min, B 100%→100%. The column temperature was maintained at 30°C, and the injection volume was 0.5 μL.

The positive ion mode was used for mass detection, and the following parameters were set: drying gas temperature, 250°C; drying gas flow rate,13 L/min; capillary voltage (Vcap), 4000 V; nebulizer pressure, 45 psig; fragmentor voltage, 125 V; sheath gas temp, 350°C; sheath gas flow, 11 L/min; and acquisition rate, 1.5 Spectra/s. The MS data were collected in the full scan mode from m/z 50–1000. The mobile phase flowed to the waste liquid in the first minute of injection.

#### 2.6.4. Metabolic profiling and principal component analysis (PCA)

The Mass Hunter Qualitative software was employed for obtaining the total ion chromatograms of both tumor group and dose group, respectively. Data were normalized by peak detection and peak alignment using the Profinder B.08.00 software, and were then converted into the.cef files. In addition, the Mass Profiler Professional (MPP) B.14.00 software was utilized for PCA, so as to discern the similarities within groups and differences between groups.

#### 2.6.5. Biomarkers identification and metabolic pathway analysis

Using the MPP software, the differences in data were statistically analyzed by T test analysis and variance analysis, with tumor group as the comparison object. In the meantime, *P* < 0.05 and *f* > 2 were used as the thresholds to screen the differences within metabolites. Moreover, the ID Browser function was employed to match the Metlin, HMDB and KEGG online libraries and to generate the molecular formula for identifying different metabolites. Thereafter, the pathway analysis function was further applied in exploring the signaling pathways affected by the differential metabolites, particularly in the KEGG database. Furthermore, the MetaboAnalyst 4.0 software (**http://www.metaboanalyst.ca**) was employed for pathway enrichment analysis to explore the potential pathways (p < 0.1) in SSE for the treatment of NSCLC.

### 2.7. Mechanism of action based on network pharmacology

#### 2.7.1. Target prediction for the absorbed ingredients of SSE

In line with the theory that active ingredients should be first of all absorbed in the body [10], the 26 prototypal components and related metabolites absorbed in rat plasma were taken as the objects of study to explore the targets. In this study, the potential targets of these compounds were predicted against the ChEMBL (**https://www.ebi.ac.uk/chembl/**), STITCH (**http://stitch.embl.de/**) and UniProt (**https://www.uniprot.org**) databases. Then, the component-target network diagram was constructed using the Cytoscape 3.7.2 software.

#### 2.7.2. Differential genes related to NSCLC regulated by the SSE ingredients

The RNAseq-IlluminaHiSeq data of 576 subjects, including 517 patients with lung adenocarcinoma and 59 healthy subjects, were collected from TCGA database (**http://cancergenome.nih.gov/**) and UCSC Xena database (**https://xena.ucsc.edu/**). Later, the above data were analyzed with Tpm correction and p-value adjustment, using the Perl and edgeR software packages (Bioconductor), so as to interpret the matrix of the differentially expressed genes (DEGs, fold change > 1.5 and p < 0.05). Thereafter, the genes regulated by the SSE components and the disease differential genes were merged to obtain their common genes. Afterwards, the component-target network diagram was established by the Cytoscape 3.7.2 software.

#### 2.7.3. Validation of the potential metabolic pathways

The NSCLC-related DEGs regulated by the SSE ingredients were imported into the KEGG online database to verify their effects on the pathways identified from cell metabonomics research from a clinical point of view, and the relationships were visualized by using the Cytoscape 3.7.2 software.

## 3. Results

### 3.1. Chemical components absorbed in rat plasma

**Fig 1:**
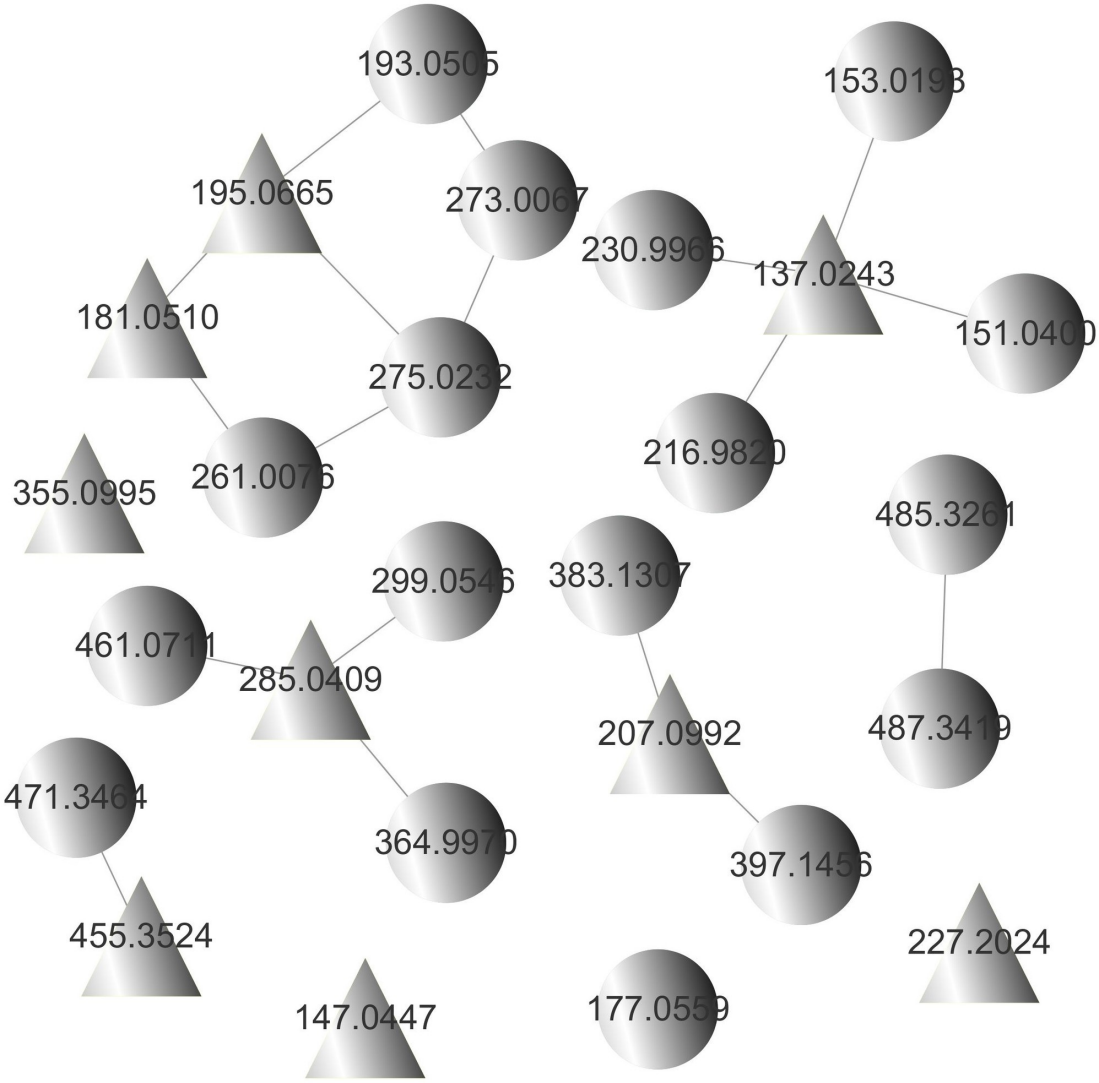
Molecular network of drug-containing plasma in rats. (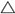, prototypal compounds; 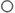, related metabolites).

**Fig 2:**
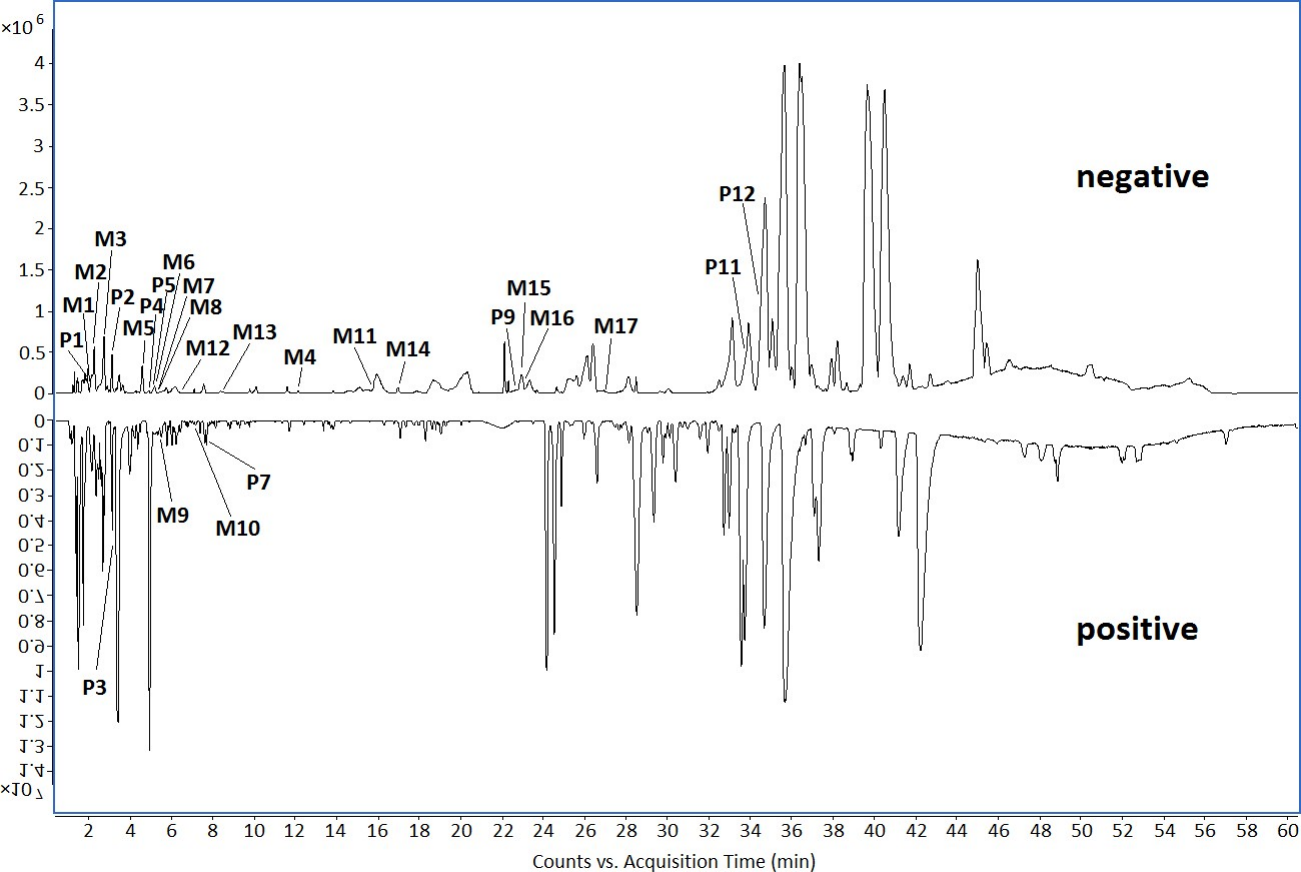
The base peak chromatogram (BPC) of chemical components absorbed in rat plasma in both negative and positive modes.

By constructing the molecular network of drug-containing plasma in rats (Fig 1), combined with the analysis results of chemical components in SSE in our previous study [6], a total of 26 compounds were identified in rat plasma, including 9 prototypal components and 17 related metabolites (Table 1, Fig 2). The absorbed prototypal components were then subjected to oxidation, reduction, methylation, sulfation, glucuronidation, and sulfation combined with methylation.

**Table 1:** Chemical components absorbed in rat plasma.

No.	Retention Time (RT)(min)	Formula	Ion type	Calculated Mass (Da)	Theoretical Mass (Da)	Mass Error (ppm)	Fragmentation	Identified prototype compounds	Metabolites
P1	2.159	C_7_H_6_O_3_	[M-H]^-^	137.0244	137.0243	-0.73	108	Protocatechuic aldehyde	
M1	2.192	C_7_H_6_O_6_S	[M-H]^-^	216.9812	216.9820	3.69	137		Protocatechuic aldehydesulfate
M2	2.671	C_8_H_8_O_6_S	[M-H]^-^	230.9969	230.9966	-1.30	137		Methyl protocatechuic aldehydesulfate
M3	2.985	C_7_H_6_O_4_	[M-H]^-^	153.0193	153.0193	0.00	109		Protocatechuic aldehyde oxide
M4	12.522	C_8_H_8_O_3_	[M-H]^-^	151.0401	151.0400	-0.66	107		Methyl protocatechuic aldehyde
P2	3.150	C_10_H_12_O_4_	[M-H]^-^	195.0663	195.0665	1.03	195	Dihydroferulic acid	
M5	4.787	C_10_H_12_O_7_S	[M-H]^-^	275.0231	275.0232	0.36	195		Dihydroferulic acid sulfate
P3	3.606	C_16_H_18_O_9_	[M+H]^+^	355.1024	355.0995	-8.17	192	Cryptochlorogenic acid	
P4	4.902	C_9_H_8_O_2_	[M-H]^-^	147.0452	147.0447	-3.40	119	trans-Cinnamic acid	
P5	5.150	C_9_H_10_O_4_	[M-H]^-^	181.0506	181.0510	2.21	108	Dihydrocaffeic acid	
M6	5.167	C_9_H_10_O_7_S	[M-H]^-^	261.0074	261.0076	0.77	181		Dihydrocaffeic acidsulfate
P6								Caffeic acid	
M7	5.462	C_10_H_10_O_4_	[M-H]^-^	193.0507	193.0505	-1.04	178,149		Methyl caffeic acid
M8	5.481	C_10_H_10_O_7_S	[M-H]^-^	273.0075	273.0067	-2.93	193		Methyl caffeic acid sulfate
P7	7.684	C_10_H_16_O_3_	[M+Na]^+^	207.0992	207.0992	0.00	170,167	Schizonodiol	
M9	5.783	C_16_H_24_O_9_	[M+Na]^+^	383.1313	383.1307	-1.57	207		Schizonodiolglucuronide
M10	7.122	C_17_H_26_O_9_	[M+Na]^+^	397.1469	397.1456	-3.27	221		Methyl schizonodiolglucuronide
P8								p-Coumaric acid	
M11	15.812	C_10_H_10_O_3_	[M-H]^-^	177.0558	177.0559	0.56	133		Methyl p-Coumaric acid
P9	22.529	C_15_H_10_O_6_	[M-H]^-^	285.0405	285.0409	1.40	151,133	Luteolin	
M12	6.638	C_21_H_18_O_12_	[M-H]^-^	461.0726	461.0711	-3.25	285,151,133		Luteolin glucuronide
M13	8.274	C_15_H_10_O_9_S	[M-H]^-^	364.9973	364.9970	-0.82	285,151,133		Luteolin sulfate
M14	17.382	C_16_H_12_O_6_	[M-H]^-^	299.0562	299.0546	-5.35	285,151,133		Methyl luteolin
P10								Maslinic acid	
M15	23.002	C_30_H_48_O_5_	[M-H]^-^	487.3429	487.3419	-2.05	487		Maslinic acid oxide
M16	23.266	C_30_H_46_O_5_	[M-H]^-^	485.3272	485.3261	-1.85	485		Maslinic acid dehydrogenate
P11	33.895	C_14_H_28_O_2_	[M-H]^-^	227.2010	227.2024	6.16	227	Myristic acid	
P12	34.572	C_30_H_48_O_3_	[M-H]^-^	455.3531	455.3524	-1.54	455	Ursolic acid	
M17	27.382	C_30_H_48_O_4_	[M-H]^-^	471.3480	471.3464	-3.39	471		Ursolic acid oxide

### 3.2. Anti-lung cancer activity of SSE in C57BL/6 mice

**Fig 3:**
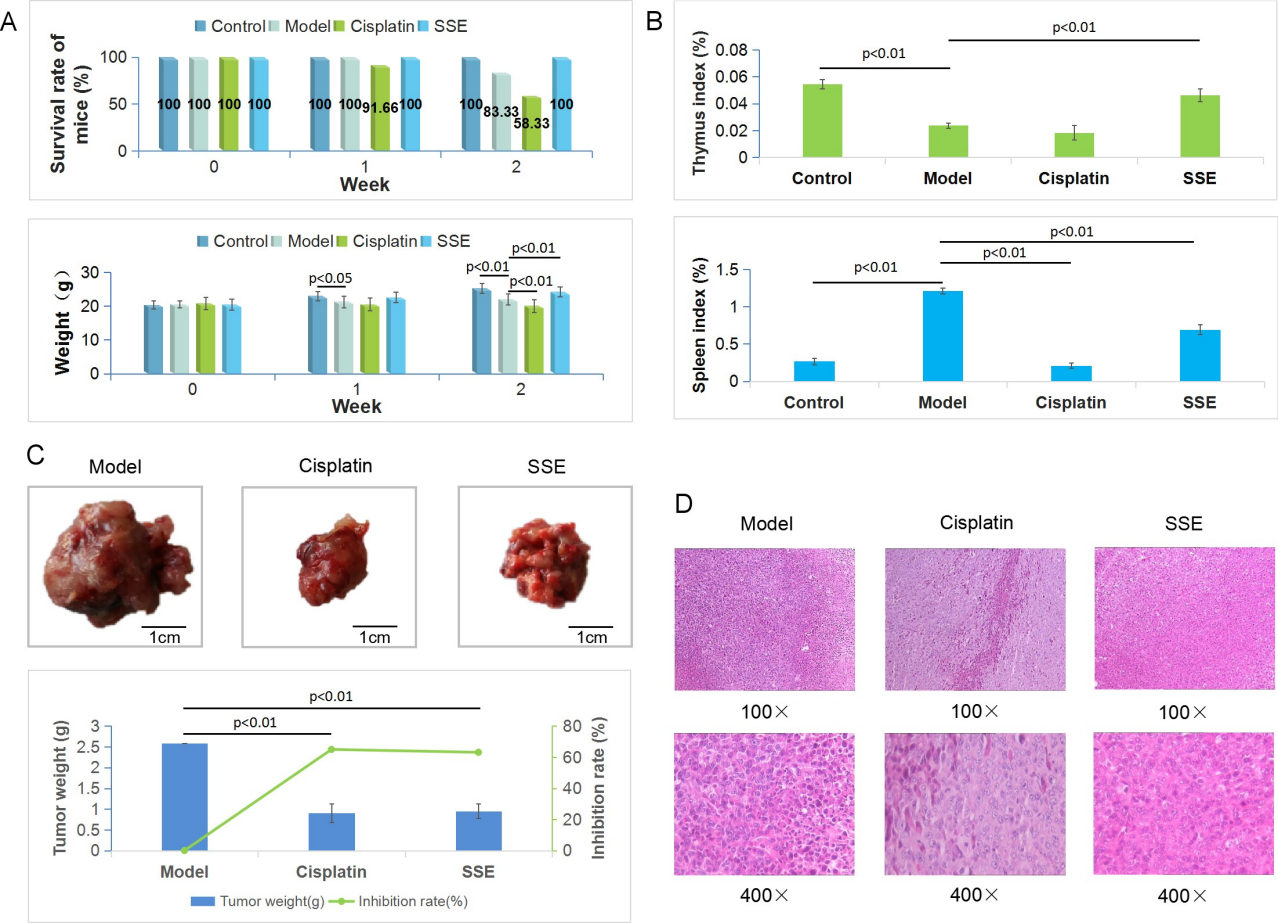
Anti-lung cancer activity of SSE in C57BL/6 mice. (A) Survival rates of mice and the changes of BW at different time points for the four groups; (B) Thymus and spleen indices of the four groups; (C) The macro views, tumor weights and inhibition rates of different groups; (D) H&E staining of tumor tissues in each group.

In terms of the survival rate, death occurred in the model group in week 2, yielding a survival rate of 83.33%; by contrast, no death was observed in mice treated with SSE, resulting in a survival rate of 100%. In cisplatin group, the survival rate was 91.66% in week 1 and 58.33% at the end of the second week. After one week of administration, the mouse BW in model group was significantly different from that of control group (p < 0.05). After 14 days, compared with model group, differences in BW of SSE group, cisplatin group and control group were all significant (p < 0.01). Besides, the mouse BW in SSE group was closer to that in control group, while that in cisplatin group was significantly reduced (Fig 3A). Besides, there were significant differences in thymus index between SSE group and control group compared with tumor group (p < 0.01), but there was no difference between cisplatin group and tumor group. Pathologically, the spleen was compensatively enlarged due to the retention of immunocytes in the spleen; as a result, the spleen index of mice in model group was the highest. By contrast, there were significant differences in control group, SSE group and cisplatin group (p < 0.01) (Fig 3B). The tumor weights in SSE group and cisplatin group were significantly lower than that in model group (p < 0.01). In addition, the tumor inhibition rate in SSE group was 63.14%, which was close to that in cisplatin group (64.99%) (Fig 3C). Moreover, H&E staining of LLC tumor tissue sections revealed that, the dense cell clusters in model group were observed to grow in nests, which were lowly differentiated, with large nuclei and abundant cytoplasm. At the high magnification, numerous pathological mitoses were observed, with no neoplastic necrosis. Additionally, apoptosis and tissue necrosis to varying degrees were observed in both SSE and cisplatin groups (Fig 3D).

### 3.3. Mechanism of action based on cell metabolomics

#### 3.3.1. Effect of SSE on the A549 cell viability

**Fig 4:**
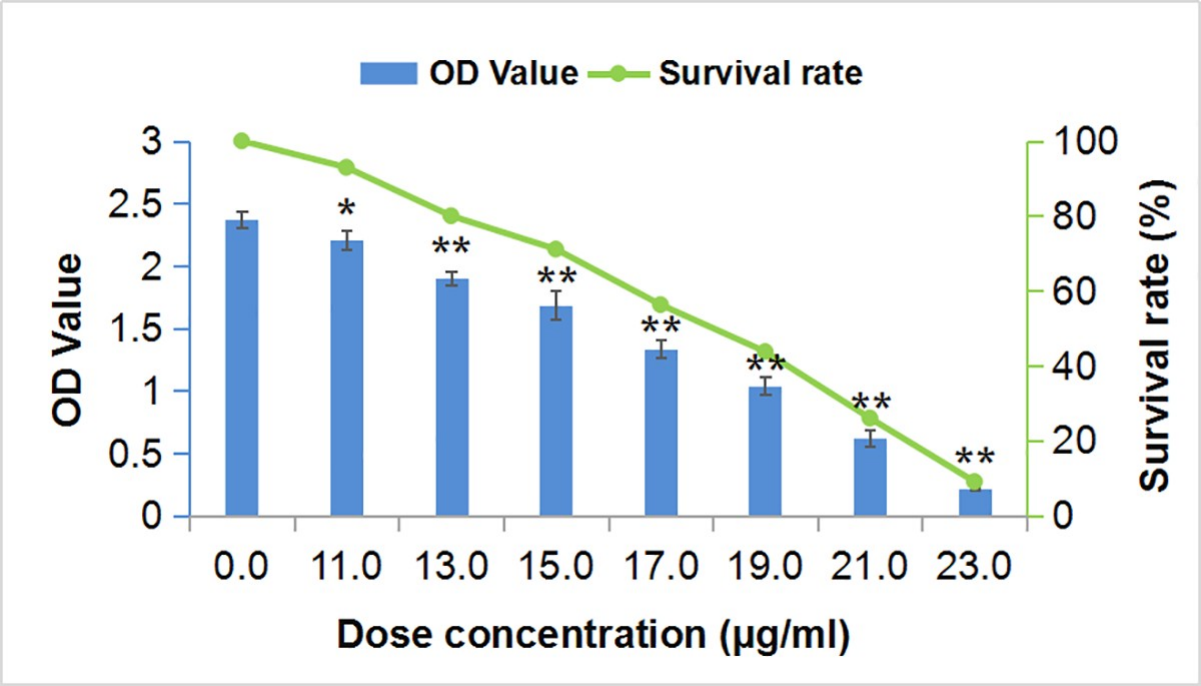
OD values and survival rates of A549 cells at different concentrations of SSE. (* p < 0.05, ** p < 0.01 compared with the concentration of 0 mg/mL).

The effect of SSE at different concentrations on the proliferation of A549 cells for 24 h was investigated (Fig 4), and the IC_*50*_ value was 17.0 μg/mL. As a result, this concentration was used in further experiments.

#### 3.3.2. Mechanism of action of SSE in treating NSCLC

**Fig 5:**
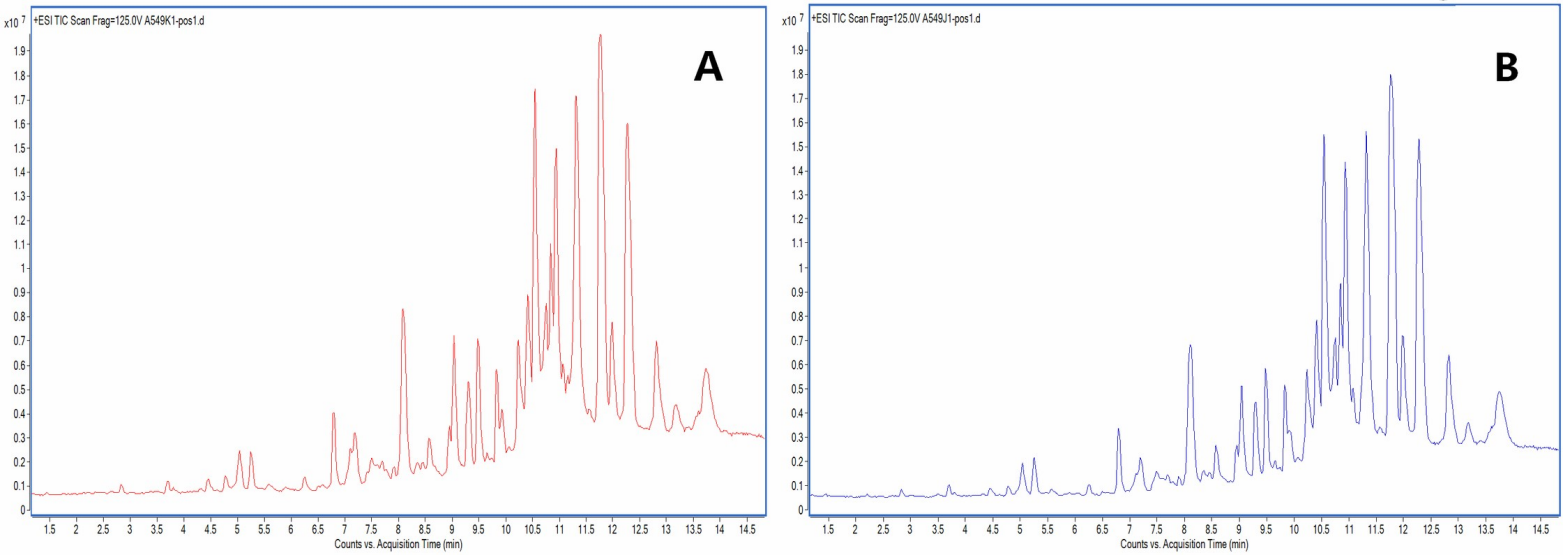
Total ion chromatograms of A549 cells. (A, tumor group, B, dose group).

**Fig 6:**
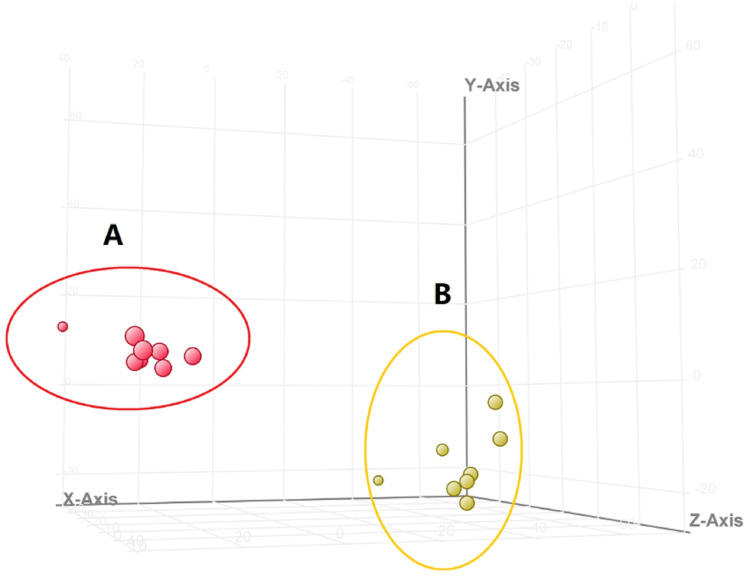
The results of PCA in the positive ion mode. (A, tumor group, B, dose group).

The total ion chromatograms of tumor group and dose group are shown in Fig 5. The results of PCA revealed significant separation between tumor group and dose group, indicating that the endogenous metabolites were significantly different between these two groups (Fig 6). As shown in Table 2, a total of 23 significantly differential endogenous metabolites were screened and identified. Compared with tumor group, GSSG, Indoleacrylic acid, 8(S)-HETE and other 12 metabolites were up-regulated in dose group, whereas Urate-3-ribonucleoside, N6-(L-1,3-Dicarboxypropyl)-L-lysine, Adenine and other 11 metabolites were down-regulated. Moreover, 16 metabolic pathways were involved, including lipid metabolism, amino acid metabolism, and carbohydrate metabolism.

**Table 2:** Differential metabolites and metabolic pathways.

No.	RT (min)	m/z	Formula	Metabolite	Trend	Pathway
1	1.390	612.1506	C_20_H_32_N_6_O_12_S_2_	GSSG	↑	Glutathione metabolism
2	3.020	187.0634	C_11_H_9_NO_2_	Indoleacrylic acid	↑	Tryptophan metabolism
3	3.402	196.1076	C_7_H_9_N_5_O	7-Aminomethyl-7-carbaguanine	↓	Folate biosynthesis
4	4.779	322.0528	C_10_H_12_N_4_O_7_	Urate-3-ribonucleoside	↓	Purine metabolism
5	6.375	293.1597	C_11_H_20_N_2_O_6_	N6-(L-1,3-Dicarboxypropyl)-L-lysine	↓	Lysine biosynthesis, Lysine degradation
6	8.551	152.0809	C_5_H_5_N_5_	Adenine	↓	Purine metabolism
7	9.312	148.0375	C_4_H_6_N_4_O	5-Aminoimidazole-4-carboxamide	↓	Purine metabolism, Histidine metabolism
8	9.841	320.2327	C_20_H_32_O_3_	8(S)-HETE	↑	Arachidonic acid metabolism
9	10.232	495.3317	C_24_H_51_NO_7_P	LysoPC(16:0)	↑	Glycerophospholipid metabolism
10	10.291	552.0914	C_14_H_23_N_3_O_15_P_2_	UDP-L-Ara4N	↓	Amino sugar and nucleotide sugar metabolism
11	10.379	162.0534	C_6_H_10_O_5_	2-Dehydro-3-deoxy-L-rhamnonate	↑	Fructose and mannose metabolism
12	10.653	368.4876	C_19_H_28_O_5_S	Dehydroepiandrosterone sulfate	↑	Steroid hormone biosynthesis
13	10.726	298.0695	C_13_H_14_O_8_	Benzoyl glucuronide	↑	Starch and sucrose metabolism, Pentose and glucuronate interconversions
14	12.137	594.1096	C_15_H_25_N_5_O_15_P_2_	Phosphoribulosylformimino-AICAR-P	↓	Histidine metabolism
15	12.887	765.5322	C_43_H_76_NO_8_P	PE(18:3(9Z,12Z,15Z)/20:2(11Z,14Z))	↓	Glycerophospholipid metabolism, Glycosylphosphatidylinositol(GPI)-anchor biosynthesis
16	12.963	791.5790	C_46_H_83_NO_7_P	PC(22:5(7Z,10Z,13Z,16Z,19Z)/P-16:0)	↑	Glycerophospholipid metabolism, Arachidonic acid metabolism, Linoleic acid metabolism
17	13.064	809.4955	C_45_H_74_NO_8_P	PE(20:3(5Z,8Z,11Z)/20:5(5Z,8Z,11Z,14Z,17Z))	↓	Glycerophospholipid metabolism, Glycosylphosphatidylinositol(GPI)-anchor biosynthesis
18	13.493	749.5350	C_43_H_76_NO_7_P	PE(22:5(7Z,10Z,13Z,16Z,19Z)/P-16:0)	↑	Glycerophospholipid metabolism, Glycosylphosphatidylinositol(GPI)-anchor biosynthesis
19	13.547	793.5965	C_46_H_85_NO_7_P	PC(20:3(8Z,11Z,14Z)/P-18:1(9Z))	↑	Glycerophospholipid metabolism, Arachidonic acid metabolism, Linoleic acid metabolism
20	13.547	809.5908	C_46_H_85_NO_8_P	PC(18:1(11Z)/20:3(5Z,8Z,11Z))	↑	Glycerophospholipid metabolism, Arachidonic acid metabolism, Linoleic acid metabolism
21	13.665	811.5091	C_45_H_76_NO_8_P	PE(20:3(5Z,8Z,11Z)/20:4(8Z,11Z,14Z,17Z))	↓	Glycerophospholipid metabolism, Glycosylphosphatidylinositol(GPI)-anchor biosynthesis
22	14.142	773.5876	C_43_H_85_NO_8_P	PC(20:1(11Z)/15:0)	↑	Glycerophospholipid metabolism, Arachidonic acid metabolism, Linoleic acid metabolism
23	14.733	789.5237	C_43_H_78_NO_8_P	PE(18:0/20:4(8Z,11Z,14Z,17Z))	↓	Glycerophospholipid metabolism, Glycosylphosphatidylinositol(GPI)-anchor biosynthesis

#### 3.3.3. Pathway enrichment analysis

**Fig 7:**
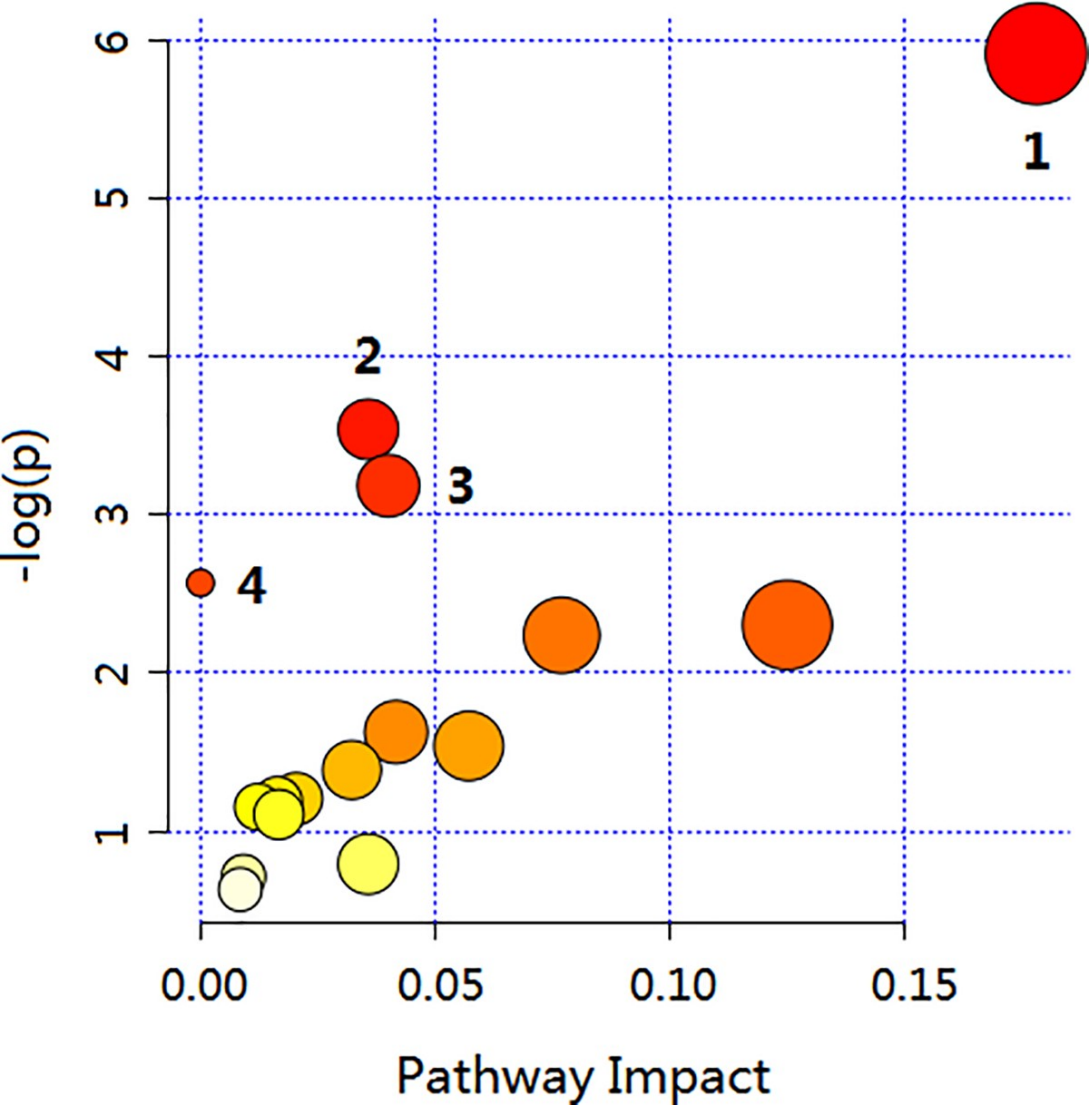
Bubble chart of pathway enrichment analysis. Each dot represents a metabolic pathway, and the label corresponds to the number of pathways in Table 3.

These 23 compounds were carried out enrichment analysis by the MetaboAnalyst 4.0 software, and it was found that these compounds were related to 16 pathways, among which, 4 (p < 0.1) were considered as the most relevant pathways involved in SSE in the treatment of NSCLC (Fig 7, Table 3).

**Table 3:** Metabolic pathways identified via the MetaboAnalyst software.

No.	Pathway Name	Total	Hits	p	Impact
1	Glycerophospholipid metabolism	39	10	0.0027133	0.17808
2	Purine metabolism	92	3	0.029078	0.03572
3	Histidine metabolism	44	2	0.041514	0.04
4	Arachidonic acid metabolism	62	5	0.076688	0

### 3.4. Mechanism of action verified by network pharmacology

#### 3.4.1. Targets regulated by the SSE components in rat plasma

**Fig 8:**
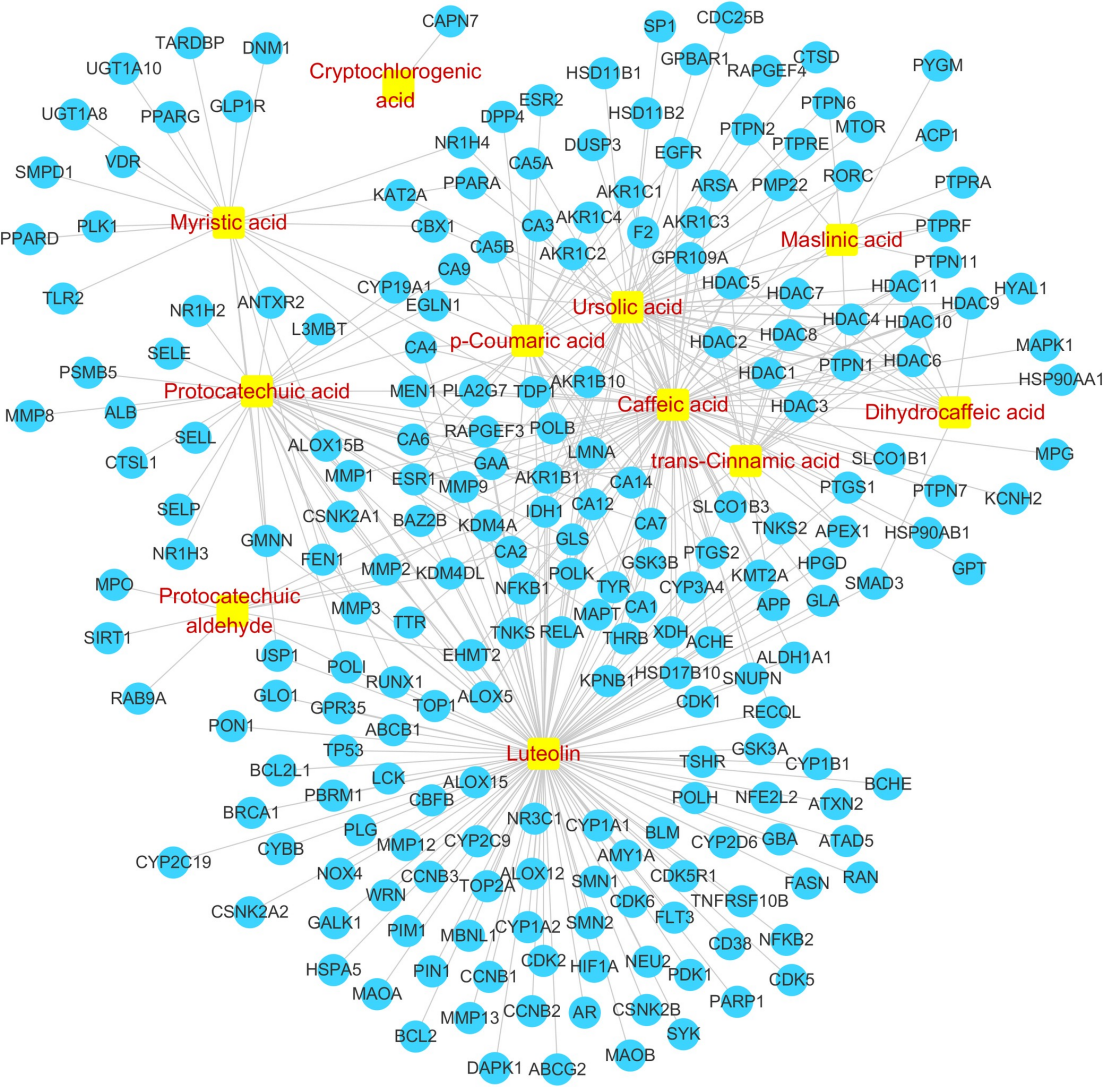
Component-target interaction network diagram. (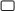, component, 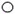, target).

By searching the ChEMBL and STITCH databases, a total of 219 human source targets regulated by the 11 prototypal components or metabolites detected in rat plasma were identified (Fig 8). Typically, protocatechuic aldehyde regulated 9 human source targets, protocatechuic acid regulated 39, cryptochlorogenic acid modulated 1, trans-cinnamic acid mediated 19, dihydrocaffeic acid modulated 12, caffeic acid modulated 84, p-coumaric acid regulated 26, luteolin modulated 130, maslinic acid regulated 9, myristic acid mediated 22, and ursolic acid regulated 55 human source targets, respectively. Fig 8 illustrates the interactions between the active compounds in SSE and the targets.

#### 3.4.2. The NSCLC-related differential genes regulated by SSE and verification of the mechanism of action

**Fig 9:**
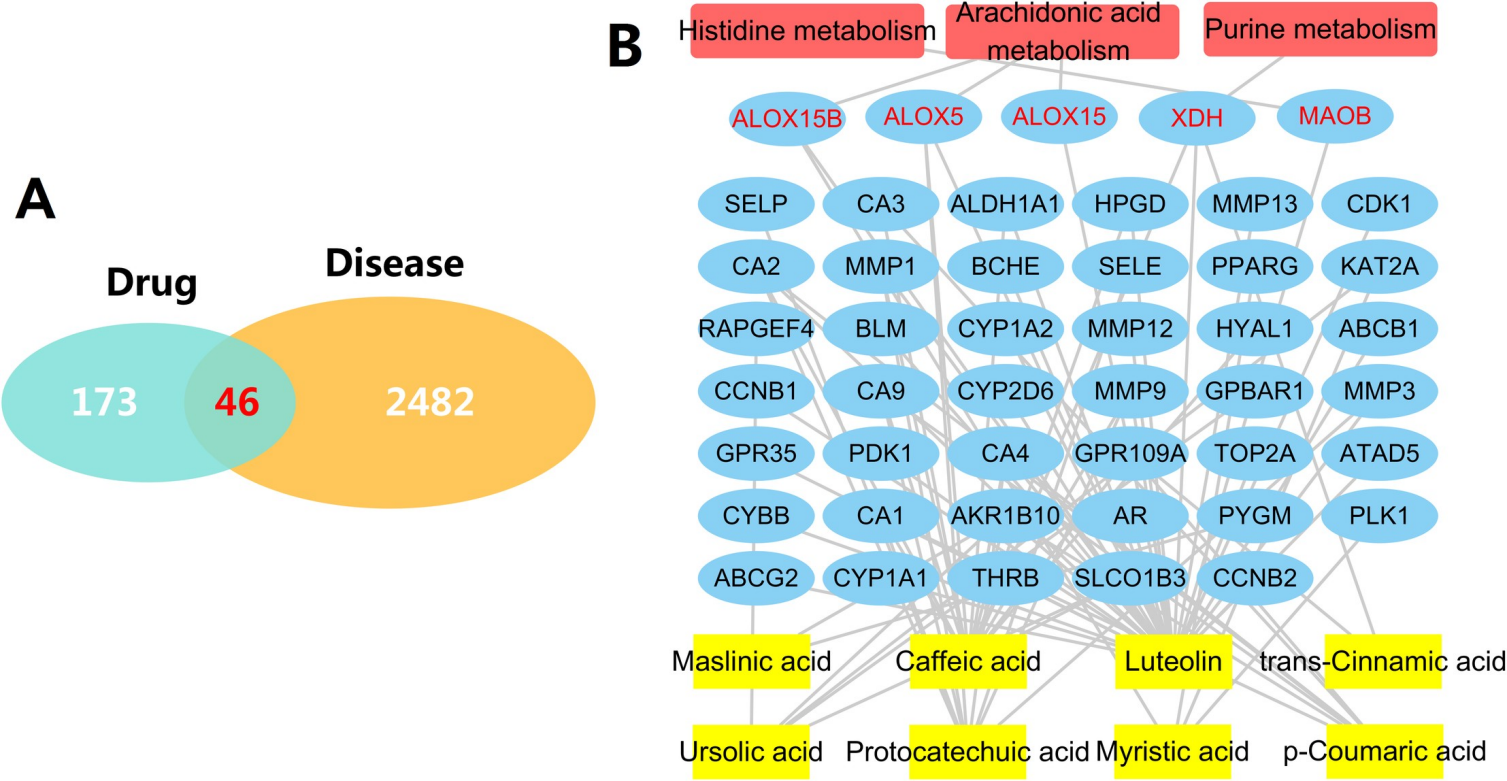
NSCLC-related differential genes regulated by SSE and component-gene-pathway interaction network diagram. (A) NSCLC-related differential genes regulated by SSE; (B) Component-gene-pathway interaction network diagram (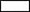, component, 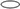, target, 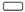, pathway, red words denote targets in the pathways).

From the differential analysis results of RNAseq-IlluminaHiSeq data based on 576 subjects in TCGA database, 2528 NSCLC-related differential genes were screened in accordance with the thresholds of p < 0.05 and logFC > 1.5. After intersecting these genes with the 219 genes regulated by SSE, a total of 46 genes were obtained (*Fig 9A*). *Fig 9B* illustrates the interactions between the active compounds in SSE and the 46 genes, and the genes interacting with the potential pathways screened by cell metabonomics, which verifies the pathways of SSE in treating NSCLC from a clinical point of view.

## 4. Discussion

Molecular network technology has only been applied in the field of TCM in recent years [14]. In this study, the molecular networking technology was utilized to identify the chemical components absorbed in rat plasma based on the GNPS database, which realizes the rapid identification and visual analysis of chemical components and their related metabolites. After intragastric administration of SSE for 1 and 2 h, the chemical components detected in the rat plasma showed no significant difference, except for their abundances. A total of 26 compounds, including 9 prototypal components and 17 related metabolites, were identified in rat plasma. These compounds included 7 phenolic acids, 1 fatty acid, 1 monoterpene, 2 triterpenoids and 1 flavonoids, whereas the related metabolites were mainly phenolic acids.

According to the secondary mass spectrometry data of each compound, the prototypal components and metabolites analyzed by this method were verified. All of the secondary fragmentation information of prototypes were consistent with the chemical composition analysis of SSE we published previously [6]. For the related metabolites, the ion peak of M1 is [M-H]^−^m/z 216.9820, the relative molecular mass is 80 (M+SO_3_) more than that of P1, and the ion peak of M2 is [M-H]^−^m/z 230.9966, 94 (M+SO_3_+CH_2_) more than that of P1. The main secondary characteristic fragment ions of M1 and M2 are all m/z 137, so M1 is speculated to be protocatechuic aldehydesulfate and M2 to be methyl protocatechuic aldehydesulfate. The relative molecular weight of M3 [M-H]^−^m/z 153.0193 is 16 (M+O) more than that of P1, and the main fragment ion m/z 109 is produced by M3 losing 1 molecule of CO_2_, so M3 is supposed to be protocatechuic aldehyde oxide (protocatechuic acid). The molecular ion peak of M4 is [M-H]^−^m/z 151.0400, 14 (M+CH_2_) more than that of P1, the loss of 1 molecule of CO_2_ produces the fragment ion m/z 107, so M4 is speculated to be methyl protocatechuic aldehyde. Similarly, the molecular weight of M5 is 80 (M+SO_3_) more than that of P2, and M6 is 80 (M+SO_3_) more than P5. The main fragment ions all present SO_3_ losing, so it is speculated that M5 is dihydroferulic acid sulfate, M6 is dihydrocaffeic acid sulfate. The molecular weight of M7 [M-H]^−^m/z 193.0505 is 14 (M+CH_2_) more than that of P6, and m/z 178, 149 are the fragment ions produced by the demethylation of M7 and the loss of one molecule of CO_2_, respectively. Therefore, M7 is supposed to be methyl caffeic acid. While the main fragment ion of M8 is m/z 193 (M+CH_2_), the molecular weight of M8 [M-H]^−^m/z 273.0067 is 94 (M+CH_2_+SO_3_) more than P6, so M8 is considered to be methyl caffeic acid sulfate. M9 removes 1 molecule glucuronic acid group obtained the fragment ion m/z 207, 14 less than the fragment ion of M10 m/z 221, and the molecular weight of M9 is 176 (M+C_6_H_8_O_6_) more, while M10 190 (M+C_6_H_8_O_6_+CH_2_) more than P7, so M9 is supposed to be schizonodiolglucuronide, and M10 to be methyl schizonodiolglucuronide. The molecular ion peak of M11 is [M-H]^−^m/z 177.0559, 14 (M+CH_2_) more than that of P8, the loss of 1 molecule of CO_2_ produces the fragment ion m/z 133, and M11 is considered to be methyl p-Coumaric acid. The molecular ion peaks of M12, M13 and M14 are [M-H]^−^m/z 461.0711, 364.9970 and 299.0546 respectively, and their relative molecular weight is 176, 80 and 14 (M+C_6_H_8_O_6_, M+SO_3_, M+CH_2_) more than that of P9. The main fragment ions m/z 285, 151, 133 are consistent with luteolin. Therefore, it is speculated that M12, M13 and M14 are luteolin glucuronide, luteolin sulfonate and methyl luteolin respectively. In addition, the molecular weight of M15 is 16 (M+O) more than P10, M16 is 2 less than M15, and M17 16 (M+O) more than P12, so M15 is supposed to be maslinic acid oxide, M16 to be maslinic acid dehydrogenate and M17 ursolic acid oxide.

In this study, the Lewis lung cancer mouse model was established to evaluate the anti-lung tumor effect of SSE from the perspectives of physiology and pathology. There were no significant differences in tumor inhibition rate and pathological sections between SSE group and cisplatin group, which proved that SSE attained comparable efficacy to cisplatin. However, from the aspects of BW, survival rate, thymus index and spleen index of mice, there were significant differences between SSE group and cisplatin group, which proved that SSE regulated the immunity of mice with lung cancer, improved their quality of life and prolonged their survival time.

To further explore the mechanism of SSE in the treatment of NSCLC, the human A549 lung tumor cells were selected and cell metabolomics was applied in analysis, which preliminarily revealed the mechanism of SSE in the treatment of NSCLC by affecting the purine metabolism, arachidonic acid metabolism, histidine metabolism and glycerophospholipid metabolism pathways. Furthermore, by network pharmacology study, it was verified that SSE regulated three pathways, including purine metabolism, arachidonic acid metabolism and histidine metabolism, to enhance the mouse immunity, improve their quality of life and prolong their survival time. It was speculated that the remarkable therapeutic effect of SSE on NSCLC might be related to its multi-target and multi-pathway therapeutic mechanism. Based on cell metabonomics study, our present study found that the glycophorphospholipid metabolic pathway has not been verified in network pharmacology research, which may be ascribed to the physiological differences between cells and human body. Therefore, the cell metabonomics-based research and verification by network pharmacology study have provided a good research direction to discover the drug mechanism in the treatment of human body diseases.

In this experiment, it was found that SSE inhibited the proliferation of lung tumor cells by regulating the purine metabolic pathway, such as 6-mercaptourine (6-MP), 6-thioguanine (6-TG), sulfomercaprine sodium and gemcitabine [16–18]. Besides, caffeic acid, ursolic acid, luteolin and trans-cinnamic acid are also the active components regulating this pathway [19–21]. Meanwhile, SSE affects the arachidonic acid metabolism pathway, like some non-steriodal anti-inflammatory drugs (NSAIDs), to achieve its anti-tumor effect [22–24]. In addition, caffeic acid, luteolin, myristic acid and protocatechuic acid are the active components regulating this pathway [25–27]. The amino acid metabolism, like the anti-tumor targeting drug L-Asparginase [28], is also closely related to tumor. SSE also affects the histidine metabolism pathway to exert its anti-tumor effect, and luteolin is identified as the active ingredient. In the next study, we will verify the specific target genes/proteins and their interactions regulated by each monomer chemical component in the above pathways by adding blockers, so as to further reveal the mechanism of SSE in the treatment of NSCLC.

## 5. Conclusions

In this study, on the basis of the clear chemical constituents of SSE, molecular networking technology based on LC-MS/MS is employed to analyze the SSE components entering the rat blood after intragastric administration. In total, 9 prototype components and 17 related metabolites are identified in the rat plasma, which reveals that caffeic acid, protocatechuic aldehyde, luteolin, ursolic acid and other components are the potential active components of SSE in the treatment of NSCLC. Using the Lewis lung cancer mouse model, it is proved that SSE not only possesses comparable anti-tumor efficacy to cisplatin, but also exerts a specific immunomodulatory effect on improving the quality of life and extending the survival time. Then, cell metabonomics is applied in combination with network pharmacology, which indicates that the effect of SSE on treating NSCLC is achieved through regulating the purine metabolism, arachidonic acid metabolism and histidine metabolism pathways.
